# The Effects of Polyphenols on Texture and Flavor of Egg Yolk: A Molecular Docking Study

**DOI:** 10.3390/foods14020295

**Published:** 2025-01-17

**Authors:** Tingting Tang, Ruyi Zhang, Cuihua Chang, Junhua Li, Luping Gu, Yanjun Yang, Yujie Su

**Affiliations:** State Key Laboratory of Food Science and Resources, School of Food Science and Technology, Jiangnan University, Wuxi 214122, China; 7220112047@stu.jiangnan.edu.cn (T.T.); 7230112055@stu.jiangnan.edu.cn (R.Z.); changcuihua@jiangnan.edu.cn (C.C.); lijunhua@jiangnan.edu.cn (J.L.); guluping@jiangnan.edu.cn (L.G.); yangyj@jiangnan.edu.cn (Y.Y.)

**Keywords:** antioxidants, egg, molecular docking, physicochemical properties

## Abstract

The effect of polyphenols on the texture and flavor of egg yolk hot gel (EY) was studied. Tea polyphenols (TP), rosmarinic acid (RE), and curcumin (CC) showed significant antioxidant properties during egg yolk processing and could effectively reduce lipid oxidation products (decreased by 68.9%, 76.4%, and 58.61%, respectively) and protein oxidation products (decreased by 47.49%, 37.47%, and 52.51%, respectively) and volatile odor components (styrene, nonanal and 1-octene-3-ol). In addition, these polyphenols enhanced the hot gel properties of the yolk, but did not significantly change the taste of the yolk. This improvement could be attributed to hydrophobic interactions, hydrogen bonds and ionic bonds formed between polyphenols and egg yolk proteins. These interactions produced a more stable structure that was less likely to unfold during heat treatment. As a result, exposure to free sulfhydryl groups, free amino acids and free fatty acids was minimized, thus reducing oxidation reactions.

## 1. Introduction

Egg yolks are a nutrient-dense food, abundant in protein, essential fatty acids, phospholipids, vitamins, and other bioactive compounds that confer numerous health benefits to humans. Constituting approximately 28–29% of the total weight of an egg, the egg yolk represents a natural supramolecular complex composed of lipids and proteins [1]. The rich diversity and abundance of proteins present in egg yolk served as a robust foundation for the formation of gel, which involved the aggregation of denatured protein molecules to create an organized network structure [2]. Upon heating, egg yolk underwent solidification or gelatinization which played a pivotal role in enhancing the texture and mouthfeel of various culinary delights such as creams, cakes, omelettes, and confectioneries while also improving their overall sensory experience [3]. Furthermore, the gel-like network structure inherent in egg yolk facilitated moisture retention and flavor encapsulation within food gels thereby imparting desired textural attributes along with enhanced flavor profiles [4]. Xie et al. through ultrasonic treatment techniques successfully improved the gelation (hardness increased from 253.5 g to 348.7 g) of egg yolk gel whereas Ren et al. reported that sodium tripolyphosphates and malic anhydride augmented the hardness by 29.73% and 56.61%, respectively [5,6]. However, despite extensive research on structural properties associated with egg yolk gels, detailed investigations pertaining to flavor enhancement in heat-induced egg yolk gels remain scarce.

Polyphenols are secondary metabolites of plants and widely found in plant components, including proteins [7]. They have a high affinity for proteins due to the presence of aromatic rings and abundant hydroxyl groups. Depending on the pH and the source of the plant protein, polyphenols interact with the covalent or non-covalent of the protein and may affect protein gelation in different ways [8]. For example, the removal of phenols from defatted flaxseed and soybeans resulted in a decrease in the storage modulus of their protein gels at pH 8, suggesting that phenols had a positive effect on gel strength [7]. The incorporation of polyphenols had been shown to improve the flavor of proteins. Diaz et al. discovered that combining whey and rice protein with blueberry polyphenols improved the sweetness and flavor of the protein [9]. In-depth understanding of how the combination of polyphenols regulates the flavor of egg yolk will enhance our understanding of the mechanism of flavor regulation of egg yolk gel and expand its application in food processing.

Therefore, our objective is to enhance the egg yolk liquid by incorporating TP, CC, and RE which have good antioxidant activity. Subsequently, we will investigate the extent of oxidation in the modified EY during heat treatment, as well as its physicochemical properties, gelling behavior, and flavor profile. The evaluation of textural and sensory attributes of the modified EY will be conducted using texture analysis, electronic nose, and gas chromatography-mass spectrometry. The molecular docking, Fourier transform infrared spectroscopy (FTIR), surface hydrophobicity measurements, and changes in free sulfhydryl groups will be employed to characterize alterations in functional groups and spatial structures. Furthermore, malondialdehyde (MDA) level, carbonyl content, free amino acid concentration, and free fatty acid composition will serve as indicators for assessing the degree of oxidation during hot gel formation. This study aims to deepen our understanding of the structure and mechanism underlying flavor release in modified EY gel while providing a theoretical basis for controlling yolk flavor.

## 2. Materials and Methods

### 2.1. Material

Eggs were purchased from the supermarket of Jiangnan University (Wuxi, China). Tea polyphenols, curcumin, and rosemary acid were obtained from Sinopharm Chemical Reagent Co. Ltd. (Shanghai, China). All reagents utilized in this study were of analytical grade unless specified otherwise.

### 2.2. Preparation of EY

The 50 mL egg yolk liquid was carefully collected in a beaker. Subsequently, TP, CC, and RE were individually added at a dosage of 0.01 g each and stirred using magnetic stirrers MMS4pro (Joanlab experiment Instrument Co., Ltd., Huzhou, Zhejiang, China) at 300 rpm for 30 min. In order to prevent water loss, the beaker was covered with aluminum foil sealing film and soaked in 90 °C water bath for 20 min to obtain the corresponding gel, named TPG, CCG, REG. Following this step, the resulting gel was cooled to room temperature and stored overnight in a refrigerator set at 4 °C. A control group without any antioxidants added served as reference, named EY.

### 2.3. Determination of Electronic Nose (E-Nose)

Samples were analyzed using a gas chromatography electronic nose Heracles II (Alpha MOS, Toulouse, France). Weigh 3 g of gel and place into a sealed 20 mL headspace vial. The test unit consisted of two columns DB-5 and DB-1701 (capillary column, 10 m × 0.18 mm) with different polarity and two FID (dihydrogen flame) detectors. For headspace extraction, the temperature was set to 60 °C, while the inlet temperature was maintained at 200 °C. The temperature program consisted of an initial increase from 50 °C to 80 °C at a rate of 1 °C per second, followed by a further increase to 250 °C at a rate of 2 °C per second.

### 2.4. Determination of Electronic Tongue (E-Tongue)

The 15 g gel was weighed, homogenized (7500 rpm, 30 s) in 100 mL distilled water, centrifuged at 4 °C and 10,000 rpm for 10 min, then the supernatant was filtered with qualitative filter paper, and the filtrate was collected for E-tongue determination. The taste analysis used a system of three taste sensors: C00 (bitter substances), AAE (umami substances), and CT0 (saltiness substances). The standard solution used in the determination process consisted of potassium chloride and tartaric acid as artificial saliva. For the purpose of negative film cleaning, a solution containing water, ethanol, and hydrogen chloride was used. For the purpose of front cleaning, a solution consisting of potassium chloride, water, ethanol, and potassium hydroxide was used.

### 2.5. Headspace Solid-Phase Microextraction Gas Chromatography-Mass Spectrometry Analysis

The volatile compounds were measured using the procedure outlined by Qi, Liu, Zhou, and Xu [10]. Take a 20 mL headspace bottle and fill it with 3 g gel. The aging extraction head was adsorbed at 60 °C for 10 min, then introduced into a gas chromatography inlet and desorbed at 250 °C for 5 min. The GC-MS analysis of volatile compounds was used the TSQ Quantum XLS instrument (Thermo, Waltham, MA, USA).

DB-WAX capillary column was used for gas chromatography analysis. The heating process consisted of an initial temperature of 40 °C for 4 min, then a heating rate of 5 °C/min to 90 °C, then a further heating rate of 10 °C/min until 230 °C was reached and held for 6 min. The mass spectrum ranged from *m*/*z* 33 to 450. The ion source temperature was set to 200 °C, the interface temperature was set to 250 °C, and the detector voltage was set to 350 V. By comparing the mass spectrum of volatile components with that of NIST14 standard library, the volatile components were preliminarily identified.

### 2.6. Texture of Samples

The texture properties of the samples were evaluated using a modified version of the method reported by Jin, Chen, Zhang, and Sheng [11]. For this assessment, a P/50 probe and a TA-XT texture analyzer (Stable Micro Systems Ltd., Godalming, UK). were utilized. The double compression TPA (Texture Profile Analysis) method was employed with specific parameters: a testing speed of 2.0 mm/s, a deformation level of 50%, and a trigger force set at 5 g. All tests were conducted at ambient temperature, and each sample was analyzed in triplicate. Key textural attributes—including hardness, springiness, chewiness, gumminess, cohesiveness, and resilience—were derived from the force–time curves obtained during the texture analysis.

### 2.7. Analysis of Surface Hydrophobicity

Protein concentrations of all samples were adjusted to 0.5 mg/mL using PBS. Fluorescence intensity was measured using a fluorescence spectrometer (Hitachi, Ltd., Tokyo, Japan), with an excitation wavelength of 374 nm and an emission spectral range of 400–600 nm. Additionally, the excitation and emission slit widths were set at 2.5 nm.

### 2.8. Analysis of Free Sulfhydryl Groups

Each gel sample (about 3 g) was diluted 10-fold with 0.04 M phosphate buffer solution (PBS, pH 7.2–7.4) and then homogenized at 12,000 rpm (2 min) using a T-18 digital homogenizer (IKA, Staufen, Germany). The homogenate was taken, centrifuged for 5000× *g* (20 min), and the supernatant was collected. The protein concentration of supernatant was determined with BCA. Subsequently, 0.02 mL of Ellman reagent was added to the mixture of supernatant (0.2 mL) and Tri-Gly buffer (2.8 mL). After 40 min of reaction in a water bath at 40 °C, the absorbance value of the sample solution at 412 nm was detected by Thermo Fisher Scientific, Thermo K3, USA. PBS was used instead of the sample solution as the blank group. Free SH content is calculated as follows:

SH (μM/g protein) = A_412_ × 73.53 × D/C

The absorbance at 412 nm (A_412_) is converted to units by multiplying by 73.53 (which derives from 106/1.36 × 104 M^−1^ cm^−1^). Here, D represents the dilution factor of 15.1, while C indicates the concentration of the supernatant in mg/mL.

### 2.9. Determination of Malondialdehyde Content (MDA)

The MDA was determined following the method of Zang et al. [12] with slight modifications. A conical flask containing 3 g gel sample was mixed with 30 mL trichloroacetic acid (TCA) mixture (37.5 g TCA and 0.5 g disodium EDTA diluted to 500 mL with deionized water) in a constant temperature water bath (SHA-B, Changzhou Jintan Jinda Instrument Co., Ltd., Jintan, China). The mixture was subjected to oscillation at 50 °C for a duration of 30 min. It was subsequently cooled to ambient temperature using an ice bath and filtered twice through double-layer filter paper to obtain the filtrate. Next, 5 mL of the filtrate was combined with 5 mL of a 0.02 M thiobarbituric acid solution and reacted in a water bath maintained at 90 °C for 30 min. In this step, TCA was utilized as a substitute for the filtrate. Following the reaction, the solution was once again cooled to room temperature via an ice bath. The absorbance of the solution at 532 nm was then measured using a UV-visible spectrophotometer (model TU-1810, manufactured by Beijing Purkinje General Instrument Co., Ltd., Beijing, China).

### 2.10. Determination of Protein Oxidation

According to the method of Hu and Xie [13], the protein carbonyl content was measured using the 2,4-dinitrophenylhydrazine (DNPH) assay. To precipitate the protein, the gel was diluted to 5 mg/mL with PBS (10 mmol/L, pH 7.0) and added with 2 mL DNPH (10 mmol/L, containing 2 mol/L HCl, Henan, China). The sample was then incubated at 25 °C for 2 h and subjected to agitation every 10 min. Subsequently, it was mixed with 40% TCA, followed by three washes and precipitations using a buffer consisting of a mixture of ethyl acetate and ethanol (1:1, *v*/*v*; 1 mL). The mixture was subjected to centrifugation at 4000× *g* for 20 min at 4 °C. The resulting pellet was then resuspended in 2 mL of 6 M guanidine hydrochloride solution (which included 10 mM PBS, pH 7.0) and incubated in a water bath at 37 °C for 20 min, with the sample being agitated every 5 min. For the control experiment, a hydrochloric acid solution lacking DNPH was utilized, and the absorbance was recorded at 370 nm. The carbonyl content was calculated with the molar extinction coefficient of 22,000 L/(mol·cm). The formula is as follows:

Carbonyl content (nmol/mg) = 45.45 × A_370_D/C

where A_370_ is the absorption value at 370 nm; D is the dilution ratio; C (mg/mL) is the concentration of the protein sample.

### 2.11. Analysis of Free Amino Acids

Free amino acids were extracted from the gel with trichloroacetic acid; 5 mL of 6% TCA solution was gently rotated with about 1 g gel for 2 min. Subsequently, the mixture was centrifuged at 4 °C and 6000× *g* for 20 min, and the supernatant was filtered through a nylon syringe filter with a pore size of 0.22 μm before being injected into HPLC for analysis. An Agilent 1260 Infinity HPLC system coupled with an Agilent 6420 triple quadrupole LC/MS system (Santa Clara, CA, USA) was employed for the analysis. Chromatographic separation was achieved on an Agilent Poroshell EC-C18 column (2.1 × 150 mm, 2.7 μm) at a flow rate of 0.3 mL/min and a column temperature of 40 °C.

### 2.12. Analysis of Free Fatty Acids

The lipid in the gel was extracted through acid hydrolysis. Following the method described by Gao et al. [14], 100 mg of lipids was dissolved in a 2% (*w*/*v*) sodium hydroxide methanol solution and heated in a water bath at 60 °C for 15 min. After cooling to room temperature, n-heptane (2 mL) was added, and anhydrous sodium sulfate was then added to the n-heptane extract for water removal. Fatty acid composition was determined using gas chromatography with flame ionization detector and Rt-2560 capillary column (100 m × 0.25 mm × 0.2 μm). The relative abundance of each fatty acid is expressed as a percentage of the total peak area.

### 2.13. Determination of FTIR

FTIR spectroscopy was acquired using Brucker Vertex 70 FTIR spectrometers (Brucker, Billerica, MA, USA). The spectral acquisition resolution was 4 cm^−1^ with an average of 32 scans.

### 2.14. Molecular Docking

To examine the interactions between phosvitin (POV) and polyphenols, molecular docking simulations were utilized. The protein structure was retrieved from the Protein Data Bank (PDB ID: 1LSH). Prior to analysis, all water molecules and ligand atoms were eliminated from the structure. The crystal structures of the flavor compounds, namely tea polyphenols (CID: 1287), curcumin (CID: 969516), and rosemary acid (CID: 5281792), were obtained from the PubChem database. Molecular docking was performed by AutoDock Vina 1.1.2 software, and the best bindings were analyzed and visualized using PLIP (Protein-Ligand Interaction Profiler).

### 2.15. Data Analysis

All quantitative analyses were conducted in a minimum of three separate experiments, with results presented as the mean ± standard deviation (SD). The comparison of means was evaluated using Duncan’s multiple range test. Statistical analyses were performed using SPSS software (version 20.0; SPSS Inc., Chicago, IL, USA). A *p*-value of less than 0.05 was considered to indicate a statistically significant difference.

## 3. Results

### 3.1. Effects of Polyphenols on Volatile Components

**Figure 1:**
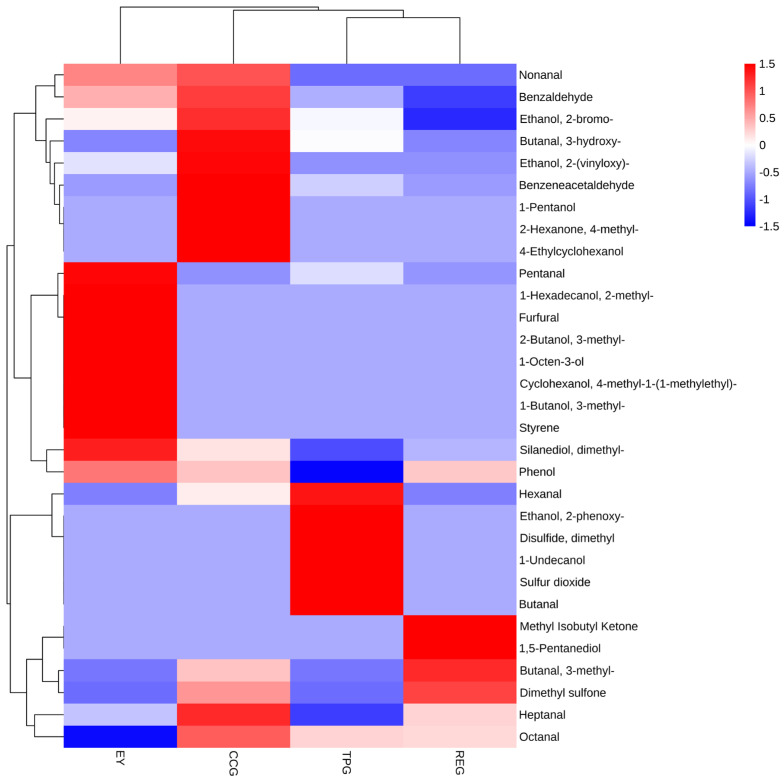
Effects of TP, CC, and RE on flavoring properties of EY.

It is well known that under heat treatment, the content of volatile flavor components such as aldehydes, ketones, and alcohols significantly increased, thereby making the aroma of EY more intense [15]. Similar findings were found in our study (Figure 1). Meanwhile, hexanal, nonanal, benzaldehyde, and 1-octene-3-ol were the main substances that caused the off-odor of EY [15]. Hexanal gave soy milk its unpleasant taste, while 1-octene-3-ol was an unsaturated alcohol with a mushroom-like flavor, and nonanal had a fatty flavor, which played a crucial role in the formation of the undesirable flavor [16]. As shown in Figure 1, the addition of TP, CC, and RE significantly reduced the levels of nonanal and 1-octene-3-ol, thereby reducing the off odor associated with modified EY. In addition, styrene also produced a pungent odor; however, the addition of three polyphenols reduced their volatility and enhanced overall olfactory experience of modified EY. The cluster analysis showed that the addition of polyphenols significantly changed the composition of volatile components in EY aroma, indicating that the characteristic flavor might change. The observed phenomena were attributed to interactions between plant polyphenols and proteins as well as carbonyl compounds, leading to the formation of a carbonyl-phenol mixture [17]. In addition, it seemed plausible that different types of polyphenols exhibited different reactivity to carbonyl compounds, resulting in different flavor substances.

### 3.2. Effects of Polyphenols on E-Nose

**Figure 2:**
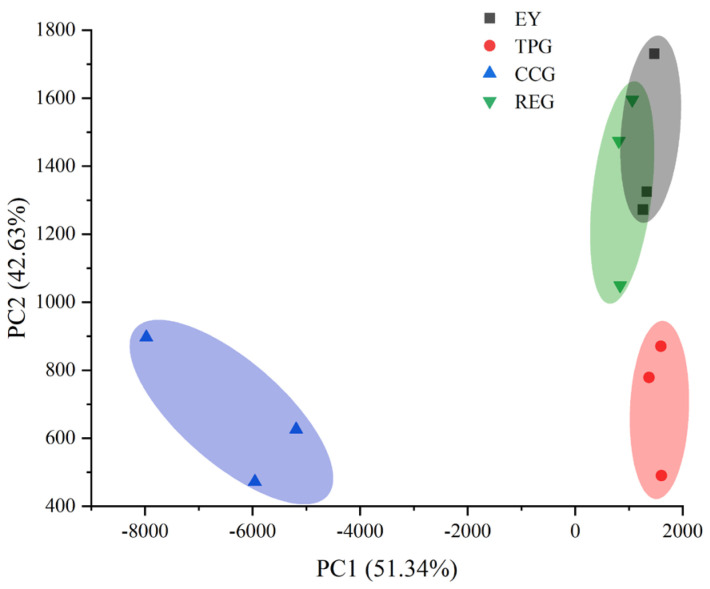
Effects of TP, CC, and RE on E-nose of EY (three points of the same color represent three repeats, and circles represent confidence intervals).

It could be seen from Figure 2 that the combined contribution rate of PC1 and PC2 is 93.97%, reflecting the overall odor profile of modified EY. The smell characteristics of EY and REG overlap, indicating that they smelled similar and did not affect consumer acceptance. The reason might be that RE could inhibit lipid and protein oxidation to the greatest extent. Moreover, the spatial structure and secondary structure of REG were similar to that of EY, which might lead to similar overall flavor of the two. In addition, CCG and TPG exhibited significantly different odor characteristics compared to other components. CC and TP had the ability to completely change the flavor of EY, possibly including the unique flavor of EY. However, GC-MS (Figure 1) showed that the addition of all three polyphenols significantly altered the volatile composition of EY. This difference might be due to the E-nose having a higher detection threshold for these flavor components, making them undetectable. Therefore, while retaining the unique flavor of EY, rosemary acid modification of egg yolk liquid was an effective way to reduce the odor of EY. Hence, while preserving the distinctive essence of EY, the modification of RE proved to be a viable approach in mitigating the off odor associated with EY.

### 3.3. Effects of Polyphenols on Electronic Tongue (E-Tongue)

**Figure 3:**
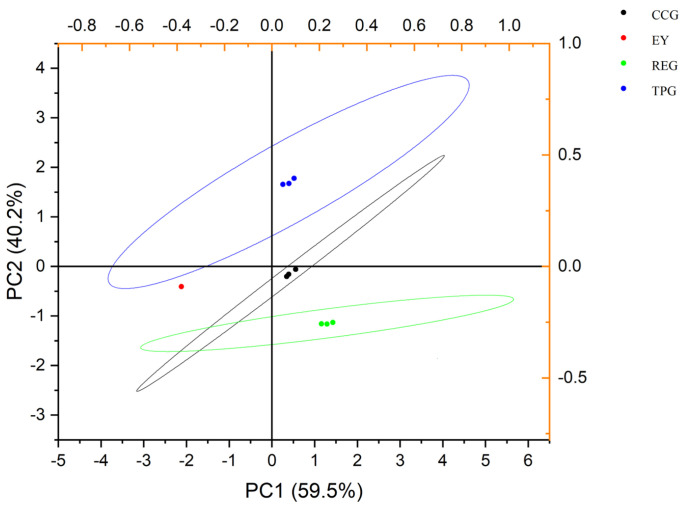
Effects of TP, CC, and RE on E-tongue of EY (three points of the same color represent three repeats, and circles represent confidence intervals).

The comprehensive taste profile of the sample can be thoroughly assessed through the use of an E-tongue. This advanced technology mimics the human taste sensation by employing a series of sensors that respond to various taste attributes such as sweetness, bitterness, sourness, saltiness, and umami. By analyzing these different components, the electronic tongue provides a detailed evaluation of the overall taste experience. As depicted in Figure 3, the combined variance of PC1 and PC2 accounted for 97.2%, indicating a high level of reliability in the results obtained. The absence of overlap among the five samples suggested distinct taste profiles, further supporting the notion that polyphenols altered the taste profile of EY, which was in accordance with the result of GC-MS. When considering findings from E-nose analyses, GC-MS measurements suggested that incorporating RE not only mitigated unwanted odors but also better preserved the overall taste quality.

### 3.4. Effects of Polyphenols on Texture

**Figure 4:**
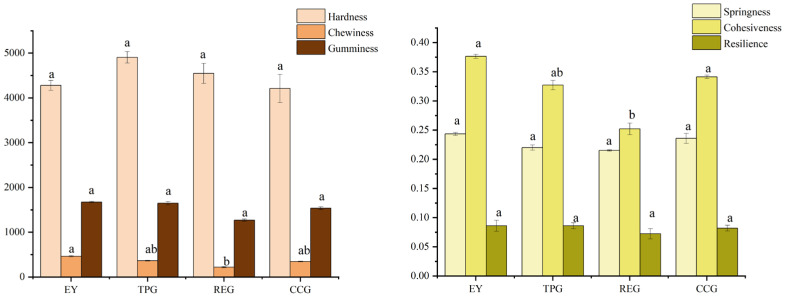
Effects of TP, CC, and RE on hardness, springiness, cohesiveness, chewiness, gumminess, and resilience of EY (different letters indicate significant differences between different polyphenols).

The formation and extension of the gel network structure significantly affected the processing performance. The protein involved in the formation of the gel network structure would change under the interference of external additives, resulting in the interference of bond maintenance in the gel network. The taste perception of the mouth directly affected the acceptance of food by consumers. Figure 4 showed the textural characteristics of the sample, including hardness, springiness, chewiness, gumminess, cohesiveness, and resilience. Hardness plays a crucial role in influencing various textural properties of food, including gumminess, chewiness, and cohesiveness [18]. These attributes were essential in texture analysis as they directly affected the sensory experience and overall enjoyment of eating. Gumminess was specifically defined as the energy required to disintegrate a bolus of food into a state that was suitable for swallowing. This process involved multiple stages of mastication where the teeth broke down the food into smaller particles. Foods with higher gumminess required more effort to chew and break down, which could influence how satisfying or tiring the eating experience might be. Chewiness, on the other hand, referred to the amount of chewing required to break down a food item before it could be comfortably swallowed. It was closely related to the resilience of the food; chewy foods tended to resist deformation and return to their original shape after being compressed. Cohesiveness measured the internal bonding within the food matrix, indicating how well the particles sticked together during chewing. The cohesiveness of a food affected its mouthfeel and how it behaved in the mouth during consumption. As shown in the figure, the hardness and gumminess of modified EY had no significant changes compared with EY. However, the hardness of TPG was slightly enhanced because the presence of TP enhanced the three-dimensional lattice structure formed by the cross-linking of EY molecules, thus enhancing the hardness. This was consistent with other research where they found that TP enhanced the non-specific cross-linking interaction between egg white protein, increased the surface hydrophobicity, and thus formed a porous three-dimensional network microstructure [19]. However, the addition of RE reduced mastication effort and improved swallowing performance of EY, and the results obtained were in line with our expected goals. Springiness measured the ability of a gel to regain its original shape and size after deformation. Figure 4 showed that the addition of polyphenols had no notable impact on springiness or resilience, thus indicating minimal influence on EY taste perception. Cohesiveness reflected how food molecules are interconnected and represents molecular bonding strength within food products [20]. The addition of RE overall reduced the internal structural integrity of EY, but did not significantly change its texture or taste.

### 3.5. Effects of Polyphenols on Lipid Oxidation

**Figure 5:**
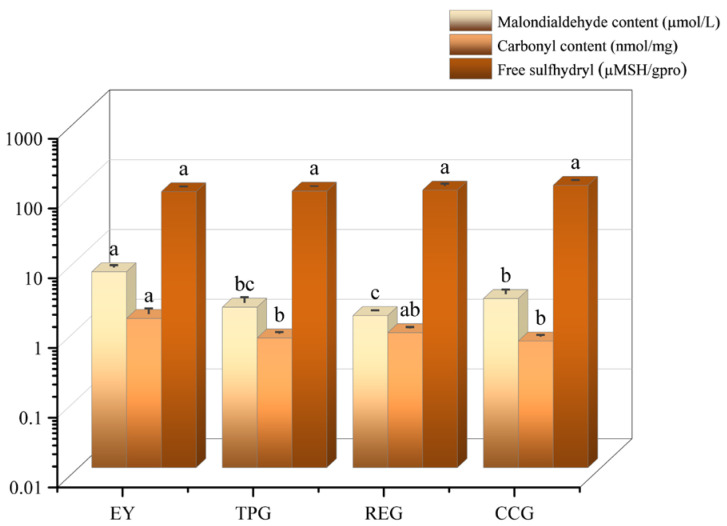
Effects of TP, CC, and RE on malondialdehyde content, carbonyl content and free sulfhydryl group of EY (different letters indicate significant differences between different polyphenols).

The determination of malondialdehyde (MDA) content was crucial because it served as a significant indicator for evaluating lipid oxidation, a process that had a profound impact on the quality of modified egg yolk gel (Figure 5). Lipid oxidation is a complex chemical reaction where fats and oils degrade over time when exposed to environmental factors such as oxygen, light, and heat. This degradation leads to the formation of various byproducts, including MDA, which is one of the most commonly used markers for assessing the extent of lipid peroxidation. The respective MDA contents were measured at 6.42, 1.99, 1.51, and 2.65 µmol/L. Notably, the addition of polyphenols resulted in a significant reduction in MDA compared to EY alone. This observation underscored the effective role of TP, CC, and RE in delaying lipid oxidation during heat induction while simultaneously enhancing overall quality. Among these polyphenols, RE exhibited the most pronounced inhibitory effect on lipid oxidation while curcumin demonstrated relatively weaker inhibition capabilities. The molecular weight of RE was at least 176.124 Da, which was smaller than CC and TP, thereby resulting in a higher abundance of RE molecules within the same mass of substances. Consequently, these RE molecules possessed an increased number of dihydroxyl structures capable of scavenging media free radicals, as well as an enriched presence of double bonds and carbonyl group couplings within their molecular framework [21]. The hot gel process facilitated the enhanced binding with EY, thereby mitigating the oxidation of EY and minimizing MDA content. The hydrophobic polyphenol curcumin had a lower water solubility and phenolic hydroxyl content compared to RE and TP, which might account for its relatively diminished antioxidant effect [22]. The binding affinity of flavonoids to BSA had been demonstrated to be enhanced by a higher degree of hydroxylation [23]. Hasni et al. observed an increase in the loading efficiency of casein polyphenols with an increasing number of OH groups, following the order: catechin-epicatechin > epicatechin hingallate > EGCG [24]. In comparison to RE and TP, CC exhibited the lowest OH content, resulting in the least binding rate with EY and consequently displaying the weakest antioxidant effect.

### 3.6. Effects of Polyphenols on Protein Oxidation

The formation of carbonyl group is the product of protein oxidative decomposition, and its content is an important index to evaluate the degree of protein oxidative decomposition in modified EY. The higher the carbonyl content, the higher the oxidation degree. As shown in Figure 5, the carbonyl contents of the samples were 1.37 nmol/L, 0.72 nmol/L, 0.86 nmol/L, and 0.65 nmol/L, respectively, indicating that the addition of polyphenols alleviated the oxidation of proteins in the heat induction process of EY. This also explained why TP, RE, and CC reduced odor in EY, as natural polyphenols had high free radical absorption capacity or strong hydrogen atom supply activity, which effectively reduced protein oxidation and thus odor perception [25]. Due to the large amount of alkoxy radicals and active secondary oxidation products, such as aldehydes and ketones, the oxidation of proteins was further triggered [26]. For example, MDA could react with proteins to form Schiff bases, which could cross-link proteins and promoted their further oxidation [27]. Therefore, the trend of protein oxidation and lipid oxidation in modified EY was the same. Li, Wu, and Wu studied the effect of MDA (0–10 mmol/L) produced during storage on the digestibility of rice bran protein. They found that oxidative modification of MDA led to protein aggregation and cross-linking [28]. Jiang et al. studied the oxidation of myofibrillar protein of silver carp with low concentration of MDA solution and found that the degree of myofibrillar protein oxidation was moderate within the appropriate concentration range, which could promote protein structure stretching and expose more active sites [29]. Additionally, protein oxidation could modify the side chains and main chains of amino acids and peptides, thereby altering their functionality [30]. This was because oxidation had the potential to induce changes in cross-linking between protein molecules, which impacted their gel properties. The mild oxidative modification promoted the formation of organized protein–protein aggregates and contributed to the creation of a regular gel matrix during heating. However, excessive cross-linking might disrupt the structured network of the gel [30]. The incorporation of TP, RE, and CC reduced the degree of oxidation in EY, consequently affecting its texture to some extent.

### 3.7. Effects of Polyphenols on Free Sulfhydryl

The protein carbonyl content did not fully reflect the degree of protein oxidation. It provided information only on carbonyl derivatives and did not include additional oxidation products of cysteine residues. The sulfhydryl group of cysteine was an important site of reactive oxygen species attack, which caused oxidative modification of proteins. Therefore, the sulfhydryl oxidation state of modified EY was characterized by the determination of sulfhydryl content. As can be seen from Figure 5, the contents of free sulfhydryl groups were 90.64 µMSH/gpro, 92.34 µMSH/gpro, 95.45 µMSH/gpro, and 111.79 µMSH/gpro, respectively. The addition of polyphenols had a slight increase in free sulfhydryl levels, suggesting that the presence of polyphenols reduced the oxidation of free sulfhydryl groups to disulfide bonds and subsequently reduced their overall degree of oxidation. In addition, MDA reacted with free sulfhydryl groups to promote the formation of stable compounds and ultimately reduce the content of free sulfhydryl groups [31]. This was similar to the results of MDA and carbonyl content. In addition, the increase in free sulfhydryl groups to a certain extent indicated the increase in intermolecular hydrophobicity, making the structure of the composite gel more stable and the gel hardness slightly increased (Figure 4) [32]. The interaction between Rosaline and cysteine was the predominant bond in the conjugate, effectively obstructing the sulfhydryl group on muscle fibrin. As a result, there was a reduction in disulfide bond formation and an increase in free sulfhydryl group content [33]. These findings aligned with our research observations.

### 3.8. Effects of Polyphenols on Surface Hydrophobicity

**Figure 6:**
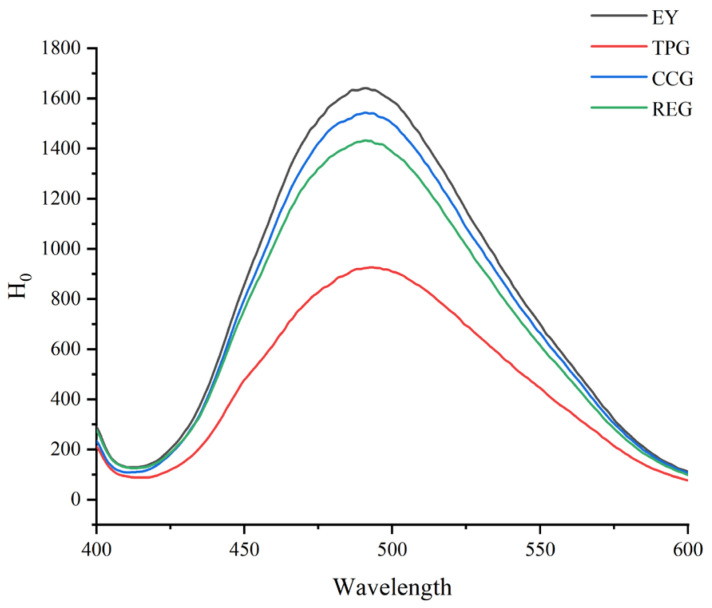
Effects of TP, CC, and RE on surface hydrophobicity of EY (the higher the peak value, the greater the surface hydrophobicity).

Surface hydrophobicity reflected the exposure degree of hydrophobic groups on the protein surface, and hydrophobic groups had a significant effect on the interface properties of proteins [34]. As shown in Figure 6, the surface hydrophobicity of modified EY was lower than that of EY, in which TPG showed a significant decrease. This suggested that TP induced significant protein aggregation. The reason might be that TP had more active phenolic hydroxyl groups, which could induce the protein to unfold, and further rearranged during the heating process, thus embedding more hydrophobic groups, and the surface hydrophobicity was lowest. Consistent with the results of carbonyl content, this was because with the increase in oxidation level the protein conformation changed, the internal fat and aromatic amino acid side chains were oxidized, the protein structure expanded, and the internal hydrophobic groups were exposed. The exposed hydrophobic groups promoted protein cross-linking through hydrophobic interactions to form oxidizing aggregates. Then the exposed hydrophobic groups were re-embedded, and the surface hydrophobicity of modified EY was finally reduced [31]. The hydrophobic flavor components (benzaldehyde, 1-octene-3-ol, nonanal) in this process might undergo hydrophobic interactions with modified EY, hindering their release and thus enhancing the flavor of EY. In addition, interactions with egg yolk proteins might prevent the indicator from binding to hydrophobic groups on the protein surface, thereby reducing surface hydrophobicity. This finding was consistent with research by Li et al., who showed that modifying meat proteins with rosmarinic acid and epicatechin reduces surface hydrophobicity [35]. The reason for lowest surface hydrophobicity of TPG lied in its high content of phenolic hydroxyl groups, which could strongly interact with EY through non-covalent binding, leading to protein aggregation and a significant reduction in oxidation degree. This finding was consistent with the observed carbonyl content. Additionally, the elevation of H0 exerted a significant impact on the gel properties of proteins. Numerous studies had demonstrated that the incorporation of polysaccharides induced alterations in protein structure, leading to exposure of hydrophobic groups and subsequent hydrophobic interactions among protein molecules, thereby further enhancing aggregation and cross-linking during gel formation [36]. Despite reducing the surface hydrophobicity of EY, the addition of TP, RE, and CC might generate a substantial number of hydrogen bonds through interaction with polyphenols, consequently augmenting the gel strength of EY.

### 3.9. Effects of Polyphenols on Protein Structure

**Figure 7:**
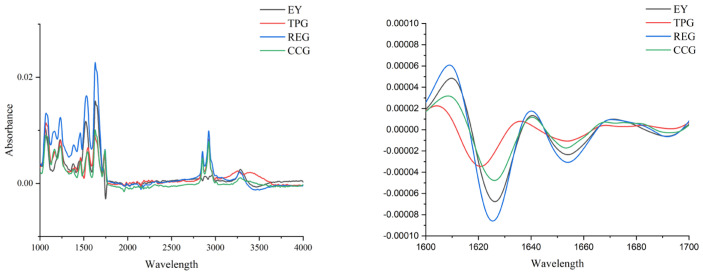
Effects of TP, CC, and RE on primary structure (**left**) and secondary structure (**right**) of EY (different absorption peaks represent different groups, as described above).

The FTIR spectra of modified EY were shown in Figure 7. Peaks observed between 3500 and 3200 cm^−1^ correspond to tensile vibrations of O-H and N-H, and the location and strength of these absorption peaks were directly related to the formation of hydrogen bonds. Notably, the absorption peak strength increased with increasing curing time, indicating a higher presence of intermolecular and intramolecular hydrogen bonds. REG showed significantly enhanced peak protein strength compared to EY, indicating a higher abundance of hydrogen bonds in this sample. Given the key role of hydrogen bonds in maintaining protein structural stability, REG had more stable structures that were less susceptible to odorous compounds induced by oxidation, which was consistent with our surface hydrophobicity results. In addition, the characteristic tensile bands at 2923 cm^−1^, 2853 cm^−1^, and 1745 cm^−1^ belong to antisymmetric tensile vibration (-CH2), symmetric tensile vibration (-CH3), and ester bond (-C=O) tensile vibration, respectively. These three bands were clearly observed in most lipid samples and protein side chains, which was consistent with the actual composition of the egg yolk [5]. It could be seen from Figure 7 that the peak intensity of EY at 1745 cm^−1^ was relatively lower than that of other components, which contradicted the expected result of carbonyl content. This inconsistency might be attributed to changes in protein structure caused by the addition of polyphenols, resulting in a decrease in carbonyl content but an increase in activity. In addition, MDA was a representative marker of lipid peroxidation products caused by oxidative damage. Due to the presence of two carbonyl groups in the molecule, it had a profound impact on protein structure and function by forming a strong covalent cross-link with the protein. Therefore, polyphenols might affect the structure of modified EY by regulating the production of MDA.

The amide I band consists of intramolecular β-sheets (1612–1642 cm^−1^), intermolecular β-sheets (1615–1625 cm^−1^), random coils (1640–1650 cm^−1^), α-helices (1651–1660 cm^−1^), and β-turns (1661–1690 cm^−1^), respectively. As shown in Figure 7, compared with EY, the peak strength of the amide I band in modified EY had significantly changed, indicating that their secondary structure was different from that of EY. The addition of CC weakened the absorption strength of amide I and made the secondary structure of the EY sample looser. It further indicated that CC interact with egg yolk protein. In REG, the absorption strength of the intramolecular and intermolecular β-sheets increased, and the strength of the α-helices and β-turns decreased, indicating that the interaction between RE and egg yolk protein made the structure denser, and it was not easy to oxidize during heat treatment, thus reducing the odor. This was consistent with the results of carbonyl group. The enhancement of the β-sheets structure was positively correlated with gel strength [37], which was one of the reasons for the increase in REG hardness. The wavelength of TPG showed a significant blue shift, indicating that the intramolecular and intermolecular β-sheets were more stable after the addition of TP and were not easily oxidized during heat treatment, thus reducing the odor.

### 3.10. Effects of Polyphenols on Free Amino Acid

**Figure 8:**
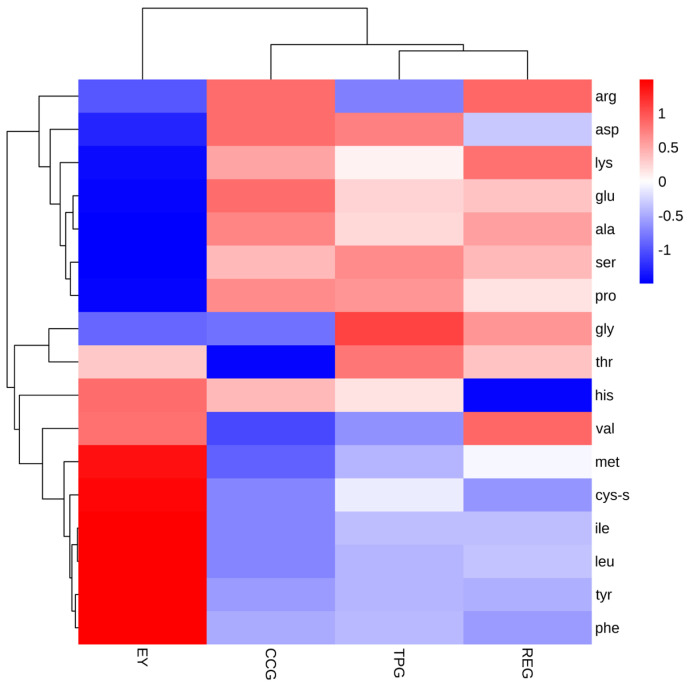
Effects of TP, CC, and RE on amino acids type and content of EY.

When a protein underwent modification, it typically resulted in alterations to the amino acid composition of the protein chain, leading to a loss of protein polymerization and/or proteolytic activity. Amongst the various amino acids, arginine, cysteine, histidine, lysine, methionine, phenylalanine, proline, tryptophan, and tyrosine were particularly susceptible to oxidation [38]. Figure 8 demonstrated that arginine, lysine, and proline content in EY was comparatively low, indicating higher levels of protein oxidation and greater consumption of these amino acids. Conversely, REG exhibited higher lysine content compared to other components suggesting lower degrees of protein oxidation resulting in fewer odor compounds. Isoleucine, leucine, and histidine were recognized as typical bitter amino acids which contributed to an unpleasant taste profile when presented at elevated levels within a product. As depicted in the figure, EY contained the highest concentrations of these three amino acids which subsequently decreased upon the addition of polyphenols, thereby decreasing bitterness perception in EY samples. Aspartic acid and glutamic acid, on the other hand, represented typical umami amino acids known for their strong synergistic effects with umami nucleotides [39]. Glutamate content was found to be highest amongst all samples examined thus potentially serving as one of the primary sources contributing towards umami flavor characteristics observed within heat-induced egg yolk gel. Furthermore, incorporation of polyphenols effectively mitigated losses associated with umami amino acids during oxidative processes thereby preserving the egg yolk umami taste profile throughout hot gel formation.

### 3.11. Effects of Polyphenols on Free Fatty Acid

**Figure 9:**
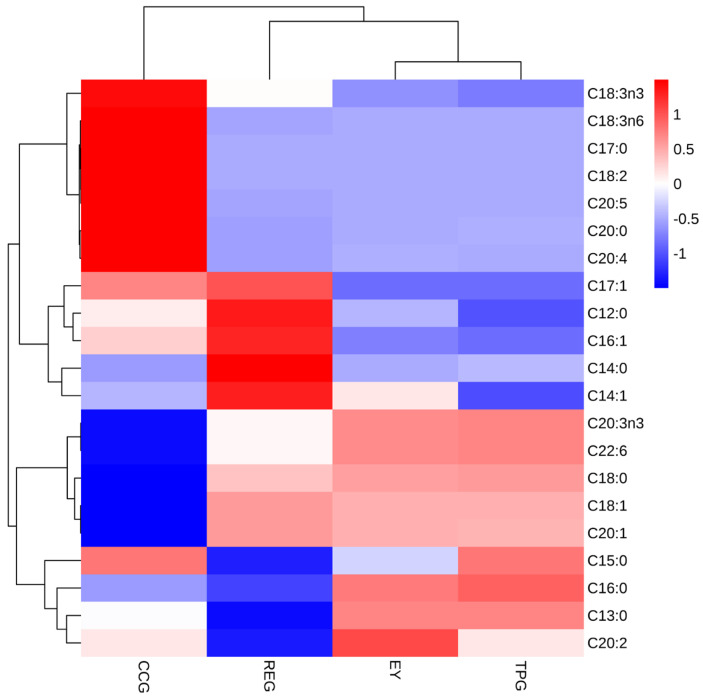
Effects of TP, CC, and RE on fatty acids type and content of EY.

The fatty acid composition in EY were crucial factors that influenced the stability of lipid oxidation. As depicted in Figure 9, a total of 21 fatty acids were identified, with a low proportion of polyunsaturated fatty acids, the highest proportion being monounsaturated fatty acids, followed by saturated fatty acids. The addition of RE and TP did not alter the fatty acid profile or cause any adverse effects on the egg yolk’s nutritional value compared to EY. However, it was evident that incorporating rosemary acid and curcumin did change the relative content of fatty acids in EY. Therefore, from a nutritional perspective, it was not recommended to add CC for improving the odor of EY. In general terms, long-chain polyunsaturated fatty acids such as eicosapentaenoic acid (EPA) and docosahexaenoic acid (DHA) were prone to degradation through lipid oxidation reactions leading to hydroperoxide formation which subsequently decomposed into various secondary oxidation products including hydrocarbons, vinylic alcohols, ketenes, and so forth, resulting in undesirable food flavor profiles [40]. It could be observed from the heat map that EPA and DHA contents were lower in REG compared to EY. These results indicated that less DHA was produced in REG during heat-induced processing, thereby reducing oxidative deterioration. This observation aligned with MDA results and served as one explanation for how RE treatment improved odor characteristics. The study conducted by Galobart et al. also revealed that the peroxide value of omega-3 unsaturated fatty acid-enriched egg yolk after spray drying was tenfold higher than that of fresh egg yolk [41], thereby providing further validation for our previous speculation.

Oleic acid (18:1) was widely recognized as a crucial precursor for the formation of hexanal and 2-amylfuran [42]. The mushroom-like, rust-like odor with a low threshold exhibited by 1-octene-3-ol could be attributed partially to the oxidation of linoleic acid (C18:2) and arachidonic acid (C20:4). As depicted in the Figure 9, it could be observed that the addition of TP did not exert a significant impact on the levels of oleic acid, linoleic acid, and arachidonic acid. This suggested that during the heat-induced process of EY, the primary odorants might arise from the oxidation of DHA. This finding aligned with the research conducted by Wang et al., which indicated that the “fishy” flavor associated with DHA-rich egg yolk, often challenging for consumers to accept, primarily stems from volatile aldehydes generated through lipid hydrolysis in egg yolk leading to the release of free fatty acids and subsequent oxidation [43].

### 3.12. Molecular Docking

**Figure 10:**
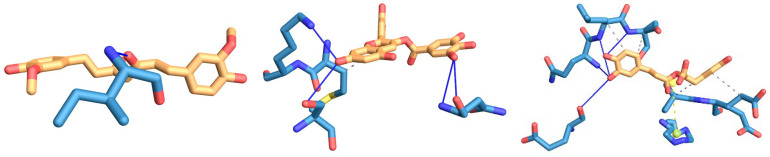
Molecular docking of CC (**left**), TP (**medium**), and RE (**right**) with phosvitin.

Molecular docking results (Figure 10) showed that the interaction sites between the three polyphenols and POV were located in the hydrophobic cavity of the protein molecule. From Table 1, we knew that CC was adjacent to the ILE residue of POV at a distance of 2.88 Å, and there was mainly hydrophobic interaction between the molecules. TP was adjacent to the LYS and isoresidues of POV, had hydrogen bond interaction force, and was adjacent to the hydrophobic residues of Leu, indicating that there was hydrophobic interaction force between them, with distances of 3.8 Å, 2.96 Å, 3.45 Å, 3.45 Å, 3.51 Å, and 3.17 Å, respectively. RE and POV formed a relatively stable conformation, with hydrophobic force and hydrogen bond interaction as the main and ionic bond interaction as the auxiliary. LEU, SER, HIS, GLU, and GLN residues of RE and POV were adjacent to each other, and hydrophobic interaction, hydrogen bond, and ionic bond occur. The distances were 3.25 Å, 3.92 Å, 3.92 Å, 3.87 Å, 3.96 Å, 3.50 Å, 4.04 Å, 3.98 Å, and 4.77 Å, respectively. This was related to the structure of polyphenols, the benzene ring of CC and TP which has high hydrophilicity, and the benzene ring of RE which has high hydrophobicity, so it can closely bind to the hydrophobic cavity of protein. In addition, the odor components in EY, such as benzaldehyde, 1-octene-3-ol, nonanal, etc., were also hydrophobic substances and could be adsorbed in the hydrophobic cavity to improve the flavor of EY. For hydrogen bond binding sites, TP and RE both contained more active hydroxyl groups, so they could react with proteins to generate a large number of hydrogen bonds, which was also the reason why TP and RE increase the hardness of EY gel.

## 4. Discussion

Egg yolks were renowned for their rich nutritional value and versatility in food processing. They served as a vital ingredient in numerous culinary applications, from baking to the production of sauces and desserts. However, one significant challenge that had limited their broader application was the development of an unpleasant odor during heat treatment, which could be off-putting to consumers. This undesirable characteristic primarily stemmed from the oxidation of lipids and proteins within the egg yolk when exposed to high temperatures. The oxidation process not only affected the flavor but also impacted the overall quality containing egg yolks. For instance, in baked goods or mayonnaise, the presence of this off flavor could significantly detract from the consumer experience. The issue aroused because heat accelerated the breakdown of fatty acids and amino acids, leading to the formation of volatile compounds responsible for the rancid smell. To address this problem, researchers had turned to natural solutions, particularly polyphenols derived from plants. Polyphenols were compounds found in various fruits, vegetables, and herbs known for their antioxidant properties. These compounds possessed a large number of active hydroxyl groups, which exhibited a strong affinity for proteins. By adding polyphenol antioxidants to egg yolks before heat treatment, it was possible to inhibit the oxidation reactions that caused the off flavors.

**Figure 11:**
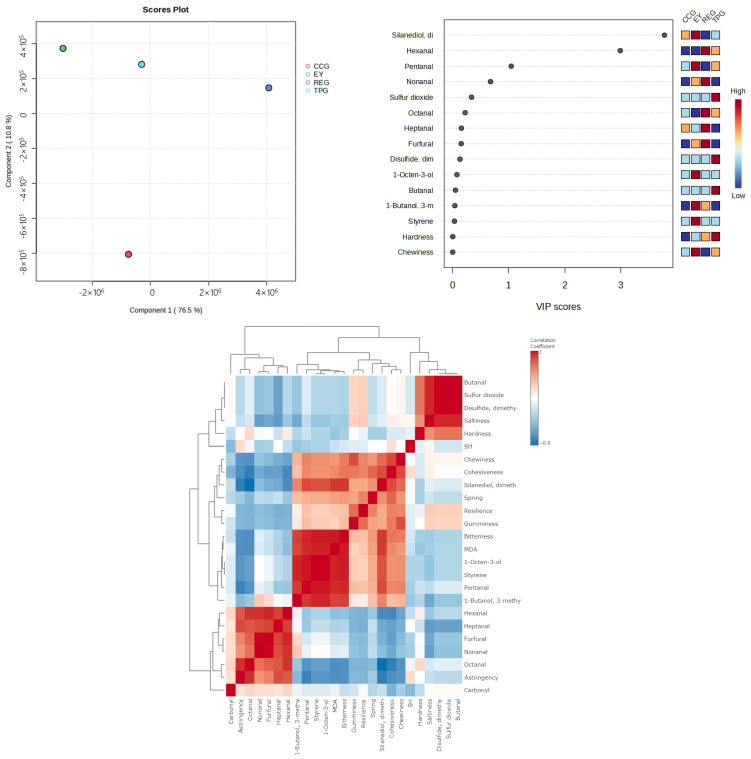
PLSDA, VIP score chart, and correlation analysis.

It can be seen from the above results that there was a correlation between the degree of egg yolk oxidation and the texture, flavor, and taste of the egg yolk. We analyzed the oxidation degree (MDA and carbonyl content), texture (hardness, springiness, cohesiveness, gumminess, chewiness, resilience), taste (bitterness, astringency, saltiness), and characteristic flavor substances (pentanal, heptanal, 1-butanol-3-methylstyrene nonanal-1-octen-3-ol, etc.) by PLSDA (partial least squares-discriminate analysis). As shown in Figure 11, the sum of C1 and C2 was 56.2%, which reflected the population distribution. The regions of EY, CCG, REG, and TPG did not overlap with each other and had significant distances between them indicating distinct differences in overall characteristics such as oxidation degree, taste, and characteristic flavor among these five samples consistent with our experimental findings. Furthermore, the modified EY was widely separated from EY indicating that the addition of polyphenols significantly altered the overall characteristics of EY. The VIP graph (Figure 11) revealed that saltiness was the main factor contributing to differences among samples in terms of sensory attributes followed by hardness, bitterness, butanal, dim sulfur dioxide, and heptanal which were greatly influenced by oxidation reactions during hot working processes. Thus, we aimed to elucidate their interrelationships through correlation analysis. The analysis of Figure 11 revealed that there was a negligible correlation between carbonyl content and the flavor and texture of EY, whereas MDA significantly impacted the flavor but had minimal influence on texture. These findings suggested that lipid oxidation primarily contributed to the undesirable flavor of EY, while having no effect on its texture. As depicted in Figure 11, MDA exhibited a significant positive association with key odoriferous compounds in modified EY, namely styrene, pentanal, 1-butanol, and 3-methyl-1-butanol. The results obtained from both MDA quantification and GC-MS analysis demonstrate that the addition of polyphenols effectively reduce MDA levels as well as the relative abundance of aforementioned flavor components. This further supported the notion that polyphenols could mitigate the off odor of EY by inhibiting lipid oxidation during thermal processing.

## 5. Conclusions

The levels of MDA and carbonyl content were found to be lower in modified EY, suggesting a reduced extent of lipid and protein oxidation compared to unmodified EY. These results indicated the excellent antioxidant properties of polyphenols during the thermal processing of egg yolk. The mechanism might be that polyphenols interacted with egg yolk proteins to promote protein aggregation and slowed protein denaturation during heating. As a result, the exposure of sulfhydryl groups, free fatty acids, and free amino acids was reduced, resulting in less oxidation. In terms of processing properties, the contents of volatile off-odor components such as styrene, nonanal, and 1-octane-3-ol in modified EY were reduced, and the gel texture and taste had no significant change compared with EY. In summary, polyphenols also had good antioxidant properties during the hot processing of EY, reducing lipid oxidation and protein oxidation and improving its hot processing properties.

The addition of polyphenol antioxidants had been shown to significantly improve the undesirable flavor that egg yolk developed after heat treatment. This enhancement was achieved without compromising the processing characteristics or overall taste of the egg yolk, which was a crucial consideration for maintaining product quality. The effectiveness of polyphenol antioxidants in inhibiting oxidation reactions during processing highlighted an important strategy for addressing the issue of off flavors in egg yolk. By mitigating these unfavorable flavors, this approach not only enhances consumer acceptance but also broadens the potential applications of egg yolk in various food products.

For instance, treated egg yolk can serve as excellent flavor enhancers in bakery items, contributing to a richer and more appealing taste profile. In addition, it can be utilized to enhance both the texture and flavor of sauce-based products, making them more palatable and desirable. The ability to preserve the natural qualities of egg yolk while improving their sensory attributes opens new possibilities for food manufacturers. It allows for greater versatility in recipe development and product innovation, ultimately leading to a wider range of high-quality food offerings that meet consumer preferences.

## Figures and Tables

**Table 1:** Molecular docking of CC, TP, and RE with phosvitin.

Protein	Polyphenols	Interaction	Amino Acid Residue	Distance
POV	CC	Hydrogen bond	ILE	2.88 Å
	TP	Hydrophobic interaction	MET	3.80 Å
		Hydrogen bond	LYS	2.96 Å
			SER	3.45 Å
			SER	3.45 Å
			ASN	3.51 Å
			ASN	3.17 Å
	RE	Hydrophobic interaction	LEU	3.25 Å
			SER	3.92 Å
			HIS	3.92 Å
			GLU	3.87 Å
		Hydrogen bond	GLU	3.96 Å
			GLN	3.50 Å
			LEU	4.04 Å
			SER	3.98 Å
		Salt bridge	HIS	4.77 Å

## Data Availability

The original contributions presented in the study are included in the article, further inquiries can be directed to the corresponding author.
